# LABCG1 Affects *Leishmania* (*Leishmania*) *Amazonensis* Infectivity but Not Virulence or Sand Fly Infection

**DOI:** 10.1021/acsinfecdis.6c00067

**Published:** 2026-05-07

**Authors:** Gustavo Rolim Barbosa, Gustavo Henrique Correa Soares, Taina Cavalcante, Pedro Cecilio, Rodrigo Pedro Soares, Paula Monalisa Nogueira, Fabiano Oliveira, Adriano Cappellazzo Coelho, Beatriz Simonsen Stolf

**Affiliations:** † Department of Parasitology, Institute of Biomedical Sciences, University of São Paulo, São Paulo 05508-000, Brazil; ‡ Laboratory of Malaria and Vector Research, National Institute of Allergy and Infectious Diseases, 2511 National Institutes of Health, Rockville 20852, Maryland, United States; § René Rachou Institute, Oswaldo Cruz Foundation, Belo Horizonte, Minas Gerais 30190-002, Brazil; ∥ Department of Animal Biology, Institute of Biology, University of Campinas, Campinas 13083-862, SP, Brazil

**Keywords:** *Leishmania* (*Leishmania*) *amazonensis*, ABCG1, PH8, CRISPR-Cas9

## Abstract

Leishmaniases are a complex spectrum of diseases caused by protozoan parasites of the genus *Leishmania*. Overall, they can be classified into two main clinical forms: tegumentary and visceral leishmaniasis (TL and VL, respectively). Our group has been studying LV79 and PH8 strains of *Leishmania* (*Leishmania*) *amazonensis*, one of the predominant TL-causing species in Brazil. PH8 is more infective *in vitro* and more virulent *in vivo* compared to LV79. Comparative proteomic analysis of promastigotes of both strains revealed that the ABCG1 transporter is 23.57-fold more abundant in PH8 than in LV79 strain. To determine whether *L. (L.) amazonensis* ABCG1 contributes to the higher virulence of PH8 strain, here we used a CRISPR-Cas9-based strategy to generate a knockout line for this gene. The mutants were compared to wild-type parasites regarding their growth, metacyclogenesis potential, LPG characteristics, resistance to complement-mediated lysis, infectivity *in vitro*, virulence *in vivo*, and ability to establish themselves in the sand fly vector. Overall, we demonstrate that while the loss of LABCG1 did not impact growth, metacyclogenesis, LPG characteristics, and resistance to complement-mediated lysis of *Leishmania* (*Leishmania*) *amazonensis* parasites, it did affect parasite infectivity *in vitro*, but not *in vivo*, both in the mouse model and in the sand fly vector. Therefore, these findings indicate that LABCG1 contributes to *L*. (*L*.) *amazonensis* infectivity but is not essential for parasite *in vivo* survival and virulence.

## Graphical abstract

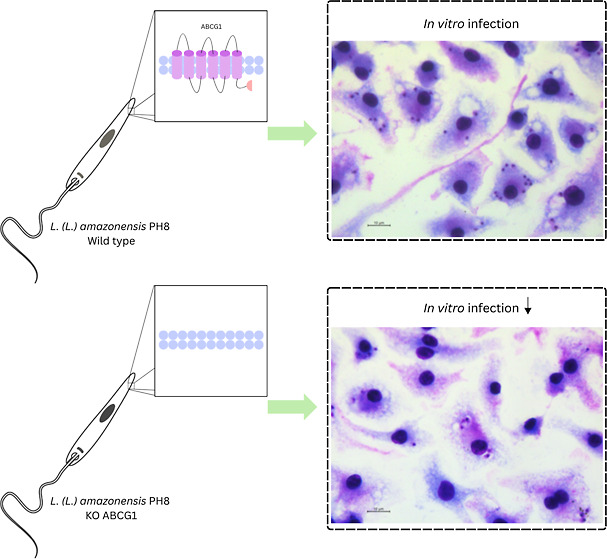

## Introduction

Leishmaniasis is a group of sand fly-borne neglected tropical diseases caused by at least 20 parasite species within the genus *Leishmania* and the subgenera *Leishmania*, *Viannia,* and *Mundinia*.^1–4^ Clinical manifestations depend on both the infecting *Leishmania* species and the host immunological background^1,5^ and can be grouped into two main clinical forms, tegumentary leishmaniasis (TL) and visceral leishmaniasis (VL). Altogether, leishmaniasis is endemic in 99 countries, of which 90 are endemic for TL and 80 for VL.^6^ The World Health Organization estimates the occurrence of 50,000 to 90,000 new cases of VL and 600,000 to 1 million new cases of TL worldwide each year.^7^ Brazil is the most endemic country for both VL and TL in South America.^7^ *Leishmania* (*Leishmania*)­*amazonensis* is one of the most important causative agents of TL in Brazil.^8^ The symptomatic infections by this species can lead to different clinical outcomes, including localized cutaneous leishmaniasis, anergic diffuse cutaneous leishmaniasis, muco-cutaneous leishmaniasis, and more rarely VL.^9^

The infective *Leishmania* metacyclic promastigotes are transmitted to a vertebrate host through the bite of a female sand fly. In the dermis of the vertebrate, parasites are mainly phagocytized by macrophages and neutrophils and differentiate into obligatory intracellular forms known as amastigotes.^10–13^ To persist within their vertebrate hosts, *Leishmania* depends on molecules known as virulence factors.^14^ A variety of these secreted or membrane-bound factors are involved in parasite internalization and immunomodulation and are potentially good targets for the development of new anti-*Leishmania* therapies.^15^ Some groups have explored proteins and LPG as virulence factors of *L*. (*L.*)­*amazonensis* by comparing two strains with distinct virulence profiles. Our group compared LV79 and PH8 strains using a proteomic approach of enriched membrane fractions and showed that the ATP Binding Cassette (ABC) transporter G1 (ABCG1) was ∼23-fold more abundant in the more virulent strain, PH8.^16^

The ABC transporter family comprises proteins present in both eukaryotes and prokaryotes.^17^ These transporters were first described in cancer cells due to their role in resistance to chemotherapy.^18^ Later, these transporters were identified in pathogenic microorganisms such as bacteria and protozoans, where they were also associated with multidrug resistance.^19^ In *Leishmania,* there are 42 ABC transporter genes divided into the ABCA to ABCI subfamilies.^20,21^ The ABCG subfamily consists of half transporters that require homo- or heterodimerization to become functional.^19^ ABCG1 was shown to be responsible for lipid transport in mammals,^22^ but its role in *Leishmania* infection is poorly understood. Indeed, the only work addressing this showed that the loss of both ABCG1 and ABCG2 in *L*. (*L*.) *major* led to differences in promastigotes’ lipophosphoglycan (LPG) composition and that stationary cultures were less efficient to undergo metacyclogenesis, indicating that the simultaneous loss of these transporters affected parasite virulence and infectivity.^23^ Interestingly, promastigotes of the PH8 and LV79 strains displayed differences in LPG composition and in resistance to complement-mediated lysis.^16,24^ We speculate that the differential expression of LABCG1 by these 2 strains is behind this phenotype.

To assess whether the abundance of *Leishmania* ABCG1 (LABCG1) in the PH8 strain contributes to its higher infectivity and virulence, we knocked out the ABCG1 gene that codes for this transporter using CRISPR-Cas9. The knockout parasites were compared to the parental lines and to controls in terms of growth, infectivity, virulence, resistance to complement-mediated lysis, LPG composition, and capacity to colonize sand flies. We also evaluated whether the LABCG1 knockout parasites were more susceptible to the drugs. Our results indicate that the loss of LABCG1 affects parasite infectivity but only in the context of *in vitro* settings.

## Results

### *L. (L.) amazonensis* PH8 and LV79 Strains Have Similar Copy Numbers of the LABCG1 Gene but Differ in RNA Levels

The much higher (23 times) abundance of LABCG1 in the proteome of membrane-enriched fractions of PH8 compared to LV79 promastigotes^16^ prompted us to investigate whether this could be related to variation on the LABCG1 gene copy or just related to differences in RNA levels. Figure 1A shows that the copy number of the LABCG1 gene in PH8 and LV79 stains is similar. On the other hand, while no differences in LABCG1 RNA levels were detected in 2 day cultures, a 2,5-fold expression difference was detected in day-4 PH8 promastigotes compared to the LV79 strain (Figure 1B). Overall, these results suggest that the transcriptional regulation of LABCG1 expression may contribute to the higher abundance of the protein in the PH8 strain.

**Figure 1:**
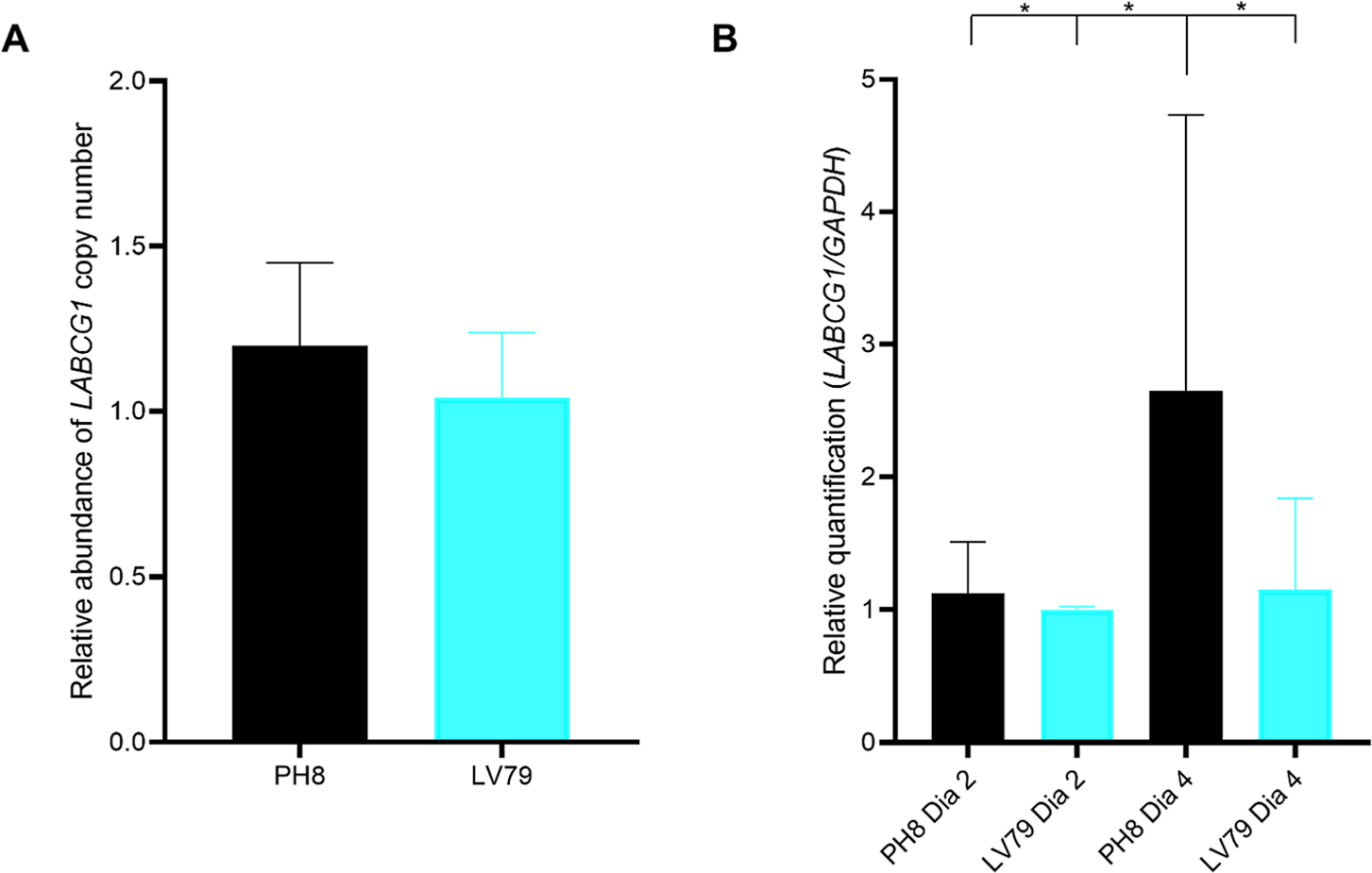
Relative quantification of LABCG1 copy number and transcript abundance. (A) Relative abundance of LABCG1 DNA in PH8 and LV79. Bars represent the ratio between the abundance of LABCG1 and G6PD. Relative abundance was determined by real-time PCR, and the graphs show the mean ± standard deviation from two independent experiments. Statistical analysis was performed using Shapiro–Wilk test, followed by the *t-*test with Welch’s correction. (B) LABCG1 transcripts abundance in promastigotes of the PH8 and LV79 strains at days 2 and 4 of culture (D2 and D4). Transcript levels were normalized to those of GAPDH, and the relative quantification was defined by the 2^–^ΔΔCt method, using the LV79 D2 data as the calibrator. Data are represented as the mean ± standard deviation from three independent experiments. Statistical analysis was performed using the one-way ANOVA with Tukey’s postanalysis (*p < 0.05).

### Generation of the *Leishmania* *(L.) amazonensis* PH8 LABCG1 Knockout and Add-Back Lines

To investigate whether the LABCG1 transporter could be related to the previously reported higher infectivity and virulence observed in PH8,^24,25^ we knocked out this gene in this strain using the CRISPR-Cas9 technology.

A PH8 line expressing Cas9 was obtained through transfection of the pTB007 plasmid^26^ and then used to replace the 2 LABCG1 alleles with puromycin and blasticidin resistance genes, as illustrated (Figure 2A) and confirmed by PCR (Figure 2B).

**Figure 2:**
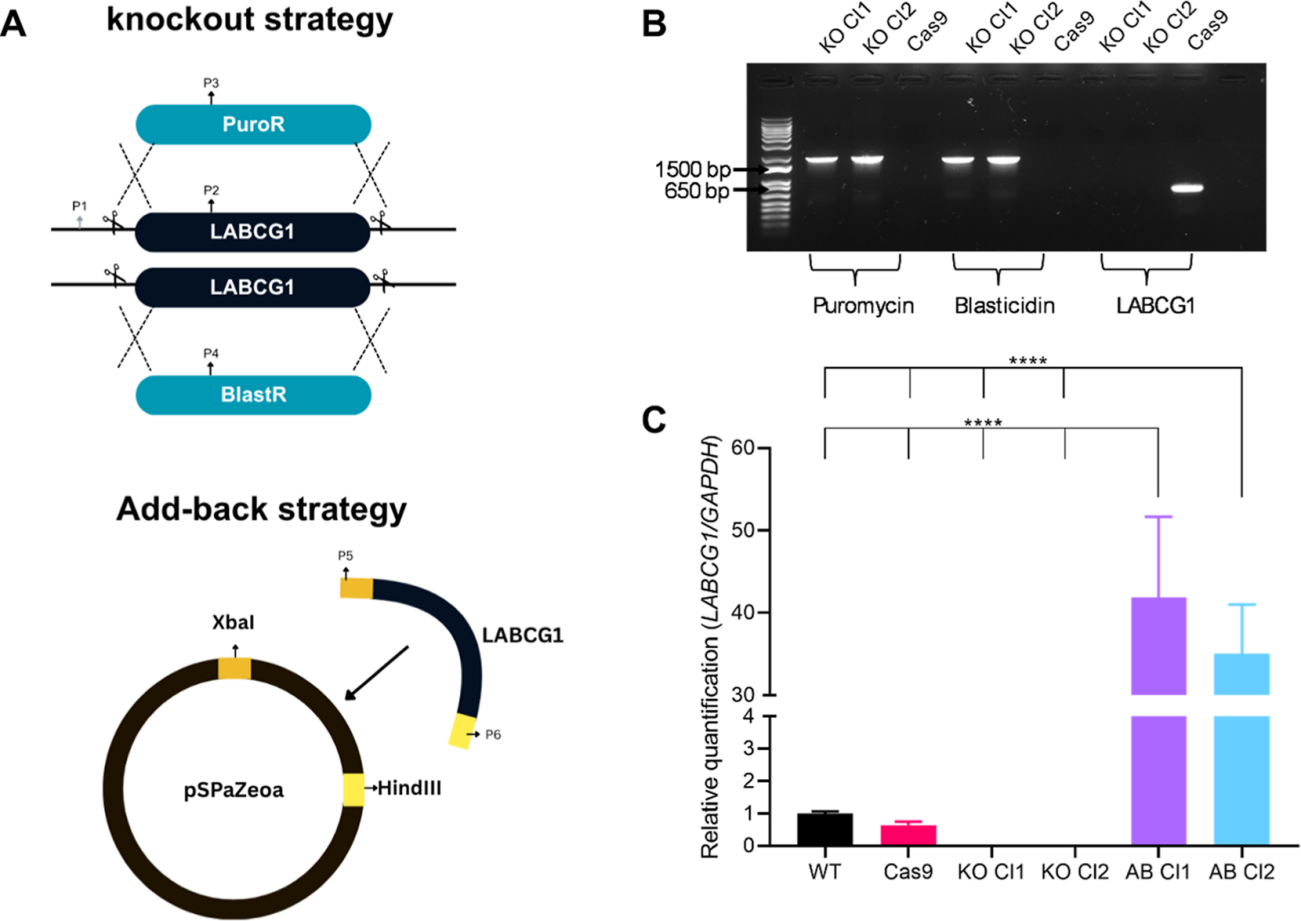
Validation of the LABCG1 mutants. (A) Strategy used to obtain and validate the LABCG1 knockout (KO) and add-back (AB) lines in the PH8 strain (Primer sequences corresponding to the black and gray arrows listed in Table S1). (B) Amplification products of the puromycin and blasticidin resistance genes and of the LABCG1 gene in KO clones 1 and 2 (Cl 1 and Cl 2) and in the control (Cas9). (C) Relative quantification of LABCG1 transcripts in promastigote cultures of the wild type (WT), Cas9, KO Cl1 and KO Cl2, AB Cl1, and AB Cl2 lines as performed by Real-Time RT-PCR. The values for each transcript were normalized to GAPDH, and the relative quantification in relation to WT parasites was determined using the formula 2^–^ΔΔCt. Mean ± standard deviation of 3 technical replicates. Statistical significance was assessed using the One-way ANOVA test with Tukey’s post hoc analysis (****p < 0.0001).

The add-back line was obtained using the strategy depicted in Figure 2A, and the sequencing of pSP72αZEOα/LABCG1 plasmid revealed a minor and probably nonrelevant substitution (Figure S1). The results shown in Figure 2C confirm that knockout parasites did not express LABCG1, while clones 1 and 2 of the add-back line (AB Cl1 and AB Cl2) showed 41-fold and 35-fold higher transcript levels, respectively, compared to wild-type PH8. Since KO Cl1 and Cl2 express no LABCG1, and AB Cl1 and Cl2 express similar levels of LABCG1 transcripts, from now on all experiments employed KO Cl1 and AB Cl1.

### LABCG1 Does Not Affect Promastigote Proliferation and Metacyclogenesis

First, all mutant and parental lines were previously used for *in vivo* infection to recover parasites with low-passage numbers. Then, we investigated whether the loss of the LABCG1 transporter could impact the parasite growth and metacyclogenesis in culture.

No differences in growth profiles were observed between the mutants and the parental PH8 strain (Figure 3A). Indeed, all lines exhibited similar growth curves, in which day 2 marked the beginning of the logarithmic phase, day 4 marked the start of the early stationary phase, and day 6 marked the late stationary phase. Moreover, while all lines showed a slight reduction in the proportion of metacyclic forms in culture, as compared to the WT (Figure 3B), this difference was not statistically significant, indicating that the loss of this transporter does not affect parasite metacyclogenesis.

**Figure 3:**
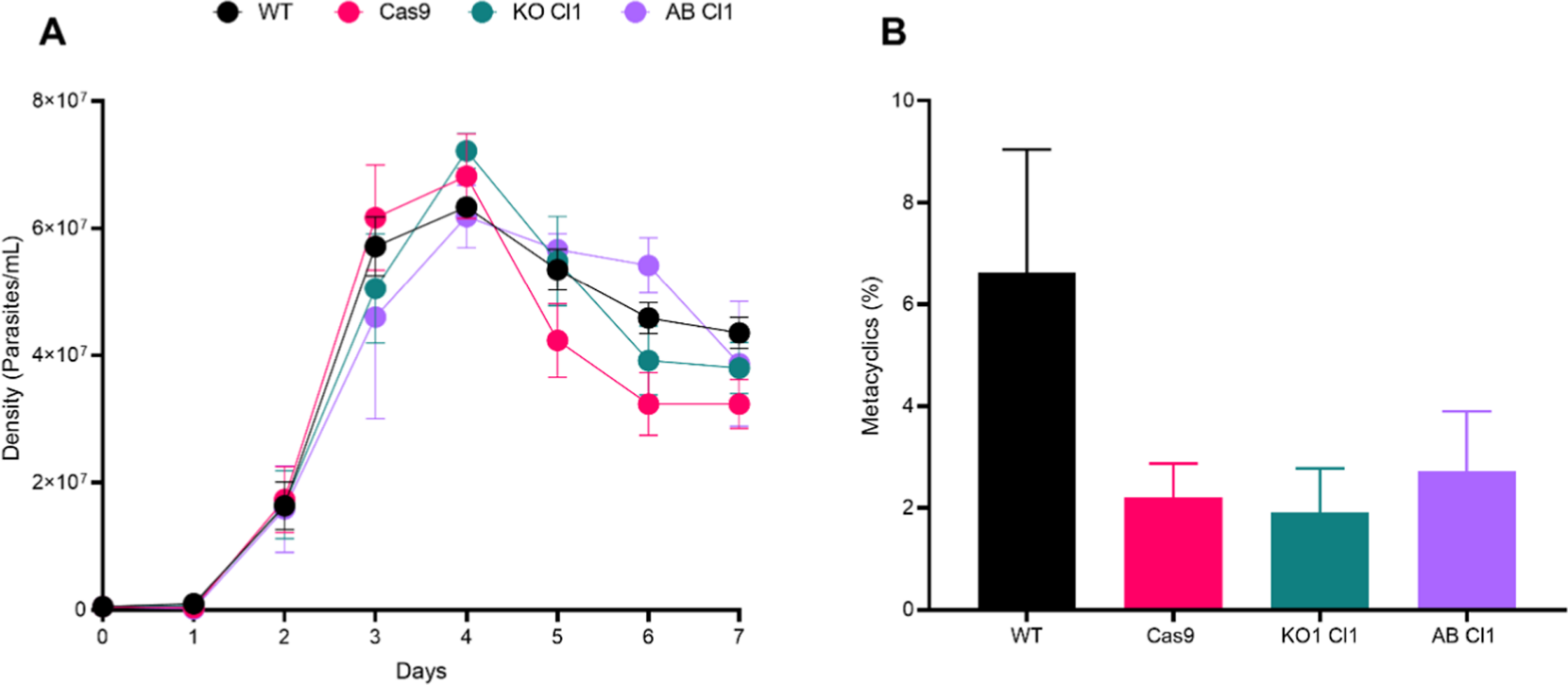
Growth curves and metacyclogenesis of WT, Cas9, KO, and AB lines. (A) Growth curves of WT, Cas9, KO Cl1, and AB Cl1 parasites. The experiment represents the mean ± standard error of three independent growth curves. (B) Percentage (%) of metacyclic promastigotes in early stationary phase cultures (Day 4) after metacyclic enrichment using the Ficoll gradient method. Data are represented as the mean ± standard error of three independent experiments. Statistical significance in B was assessed using the One-way ANOVA test with Tukey’s post hoc analysis.

### Loss of LABCG1 Impairs Parasite Infectivity In Vitro, Which Is Not Related to Differences in Phagocytosis

It was previously reported that the double loss of LABCG1 and LABCG2 in *L*. (*L*.) major impaired the *in vitro* infectivity of these parasites.^23^ To assess if the same would apply to *L. (L.) amazonensis* lacking LABCG1, infection assays were performed using bone-marrow-derived macrophages (BMDMs). Parasites lacking LABCG1 also exhibited reduced infectivity (percentage of infected macrophages) when compared with the control lines at all time points (Figure 4A). Importantly, this phenotype was reversed in add-back parasites, attesting that the loss of infectivity is indeed due to the loss of LABCG1 expression. Of note, despite the observed reduction in the percentage of infected macrophages, no significant differences in the number of amastigotes were detected among all parasite lines (Figure 4B). Representative images of the cells infected with the different parasite lines are shown in Supporting Information Figure S2.

**Figure 4:**
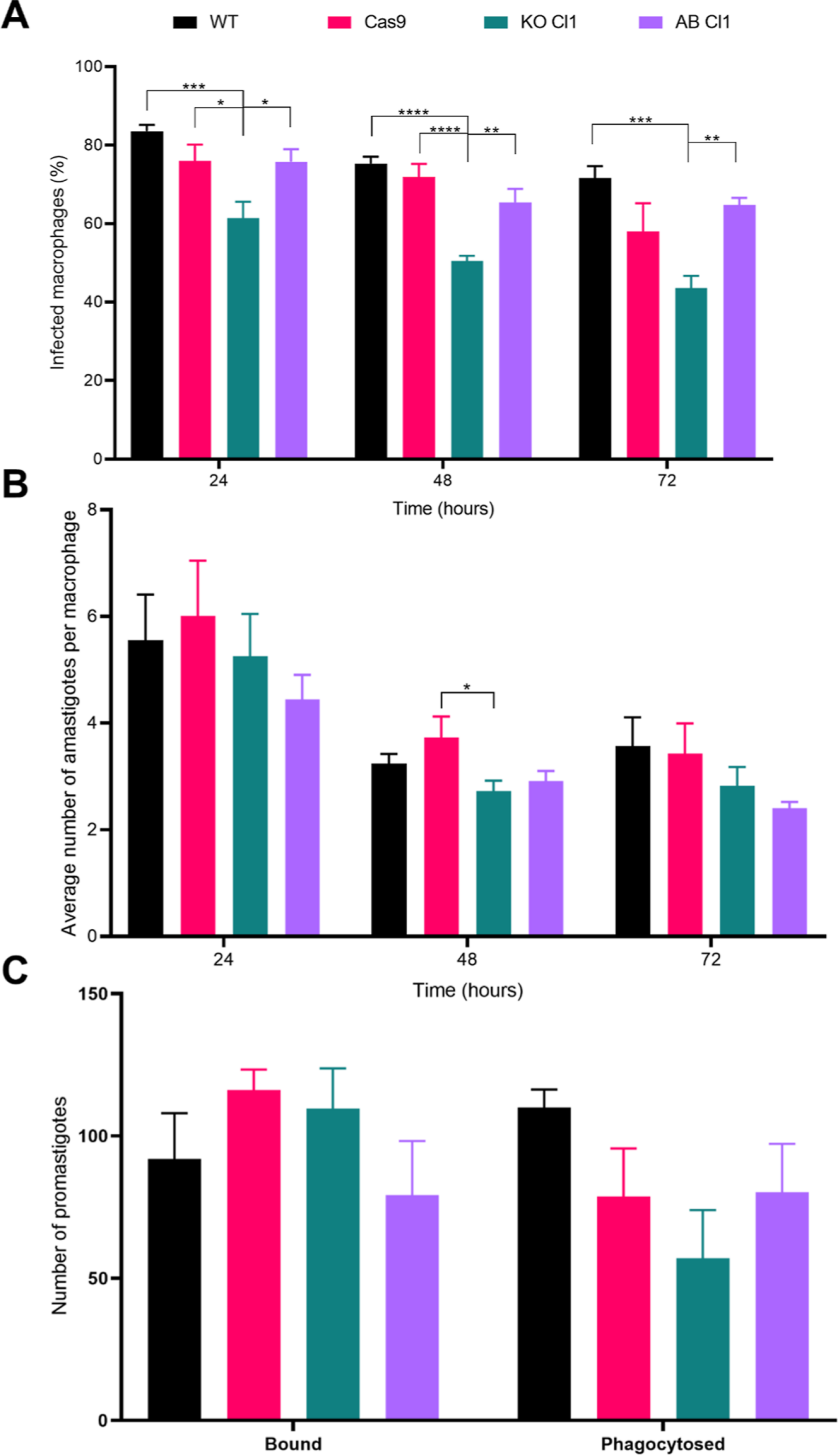
*In vitro* infection and phagocytosis of WT, Cas9, KO, and AB lines. (A) Percentage of infected macrophages (MOI 1:5) by WT, Cas9, KO Cl1, and AB Cl1. (B) Number of amastigotes per infected macrophage (MOI 1:5). Data are represented as the mean ± standard error of three independent experiments. (C) Phagocytosis assay: number of promastigotes bound and phagocytosed in 100 macrophages. Data are represented as the mean ± standard error of two independent experiments. Statistical significance was assessed using One-way ANOVA, followed by Tukey’s post hoc test. Nonsignificant data are not shown. *p ≤ 0.05; **p ≤ 0.01; ***p ≤ 0.001; ****p ≤ 0.0001. The comparisons among the *Leishmania*lines were made for each time point.

We then investigated whether the reduction in infectivity of the knockout parasites might be due to differences in phagocytosis. A phagocytosis assay was performed using BMDMs, and immunofluorescence was used to distinguish phagocytosed versus adhered parasites (images of the immunofluorescence are shown in Supporting Information Figure S3). Overall, we observed no changes in the number of phagocytosed parasites between WT and mutant lines (Figure 4C), suggesting that the lower *in vitro* virulence of *L. (L.) amazonensis* parasites lacking LABCG1 is not due to differences in the phagocytosis of these parasites.

### LABCG1 Knockout Parasites Do Not Display Differences in Resistance to Complement-Mediated Lysis and in the LPG Structure

Previous results of our group demonstrated that PH8 strain is more resistant to serum-lysis than LV79 ^24^. Therefore, next we investigated whether the LABCG1 knockout would affect parasite resistance to complement-mediated lysis. Overall, we observed that LABCG1 knockout did not impact the resistance of *L. (L.) amazonensis* promastigotes to human sera (Figure 5A,B). Of note, mouse sera led to very low and similar levels of death among parasite lines (data not shown). Unexpectedly, the add-back parasites were more sensitive to complement-mediated lysis by human sera than all other lines. We hypothesized that the re-expression of LABCG1 in the add-back parasites may somehow have affected the levels, synthesis, and composition of LPG, a molecule that plays an important role in complement resistance. However, LPGs were successfully purified and displayed no differences among lineages (WT, Cas9, KO, and AB) after immunoblotting with both antibodies, confirming previous studies with PH8 strain.^24,27,28^ *L. (L.) infantum* LPG was used as a positive and negative control for Ca7AE and LT22, respectively (Figure 5C,D) To ascertain deeper the LPG composition, repeat units were purified and subjected to Fluorophore-assisted carbohydrate electrophoresis (FACE). Consistent with previous data, FACE analysis revealed no differences in the composition of the repeat units off all lineages showing expected 2–5 β-glucosyl residues branching-off the conserved Gal­(β1,4)­Man­(α1)-PO_4_ backbone (Figure 5E). We also detected similar LPG levels in all parasite lines using flow cytometry (data not shown) of parasites. We believe that the difference observed in resistance to complement-mediated lysis in the add-back parasites is due to a mechanism not explored in this study.

**Figure 5:**
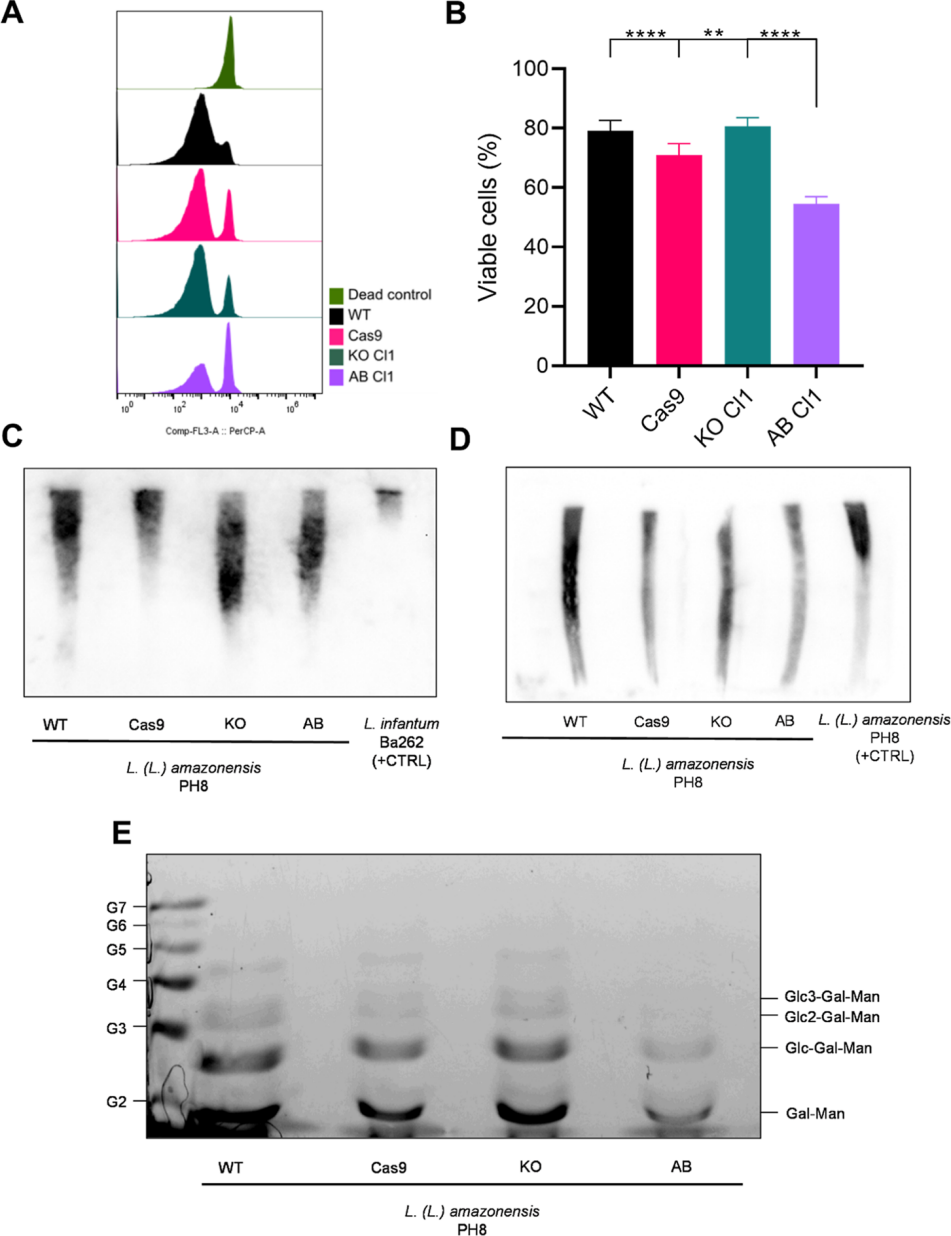
Resistance to complement-mediated lysis and LPG characteristics of WT and mutant parasites. (A) Representative histograms of serum-treated parasites (2% human serum) and dead control showing live and dead (stained with 7-AAD) parasites. (B) Viability of promastigotes (early stationary phase) treated with 2% human serum. Data are represented as the mean ± standard error of three independent experiments. Statistical significance was assessed using the One-way ANOVA test with Tukey’s post hoc analysis. **p ≤ 0.01; ****p ≤ 0.0001. (C) Immunoblotting of purified LPG from PH8 WT, mutants, and *L. (L.) infantum* Ba262 strain, probed with mAb CA7AE. (D) Immunoblotting of purified (LPG) from PH8 WT and mutants probed with mAb LT22. (E) FACE of lipophosphoglycan (LPG) repeat units from PH8 WT and mutants. Ladder in lane 1 correspond to oligoglucose representing 1–7 glucose residues (G1–G7).

### Lack of LABCG1 Does Not Impair the Ability of Promastigotes to Infect the Sand Fly Vector

We then investigated whether the lack of LABCG1 would affect parasite’s fitness within the sand fly vector. For this, we used *Lutzomyia longipalpis* sand flies, recognized as a permissive vector due to their ability to sustain infection with many *Leishmania* species, including *L. (L.) amazonensis*.^29^ Briefly, we infected *Lu. longipalpis* sand flies with the different parasite lines via artificial membrane feeding and dissected their midguts 7 days later to assess infection status. Macroscopically, no apparent differences were observed. The midguts of sand flies infected with all parasite lines showed similar features of healthy infections, including the accumulation of a dense mass of live parasites in the anterior midgut (Figure 6A; Videos S1,S2,S3 and S4). In line with this, the number of parasites per midgut of sand flies infected with LABCG1 KO parasites was similar to that recorded in sand flies infected with the control lines (WT, AB, and Cas9; Figure 6B). Overall, these results strongly suggest that the expression of LABCG1 is not essential for the establishment of *L. (L.) amazonensis* parasites in the sand fly midgut.

**Figure 6:**
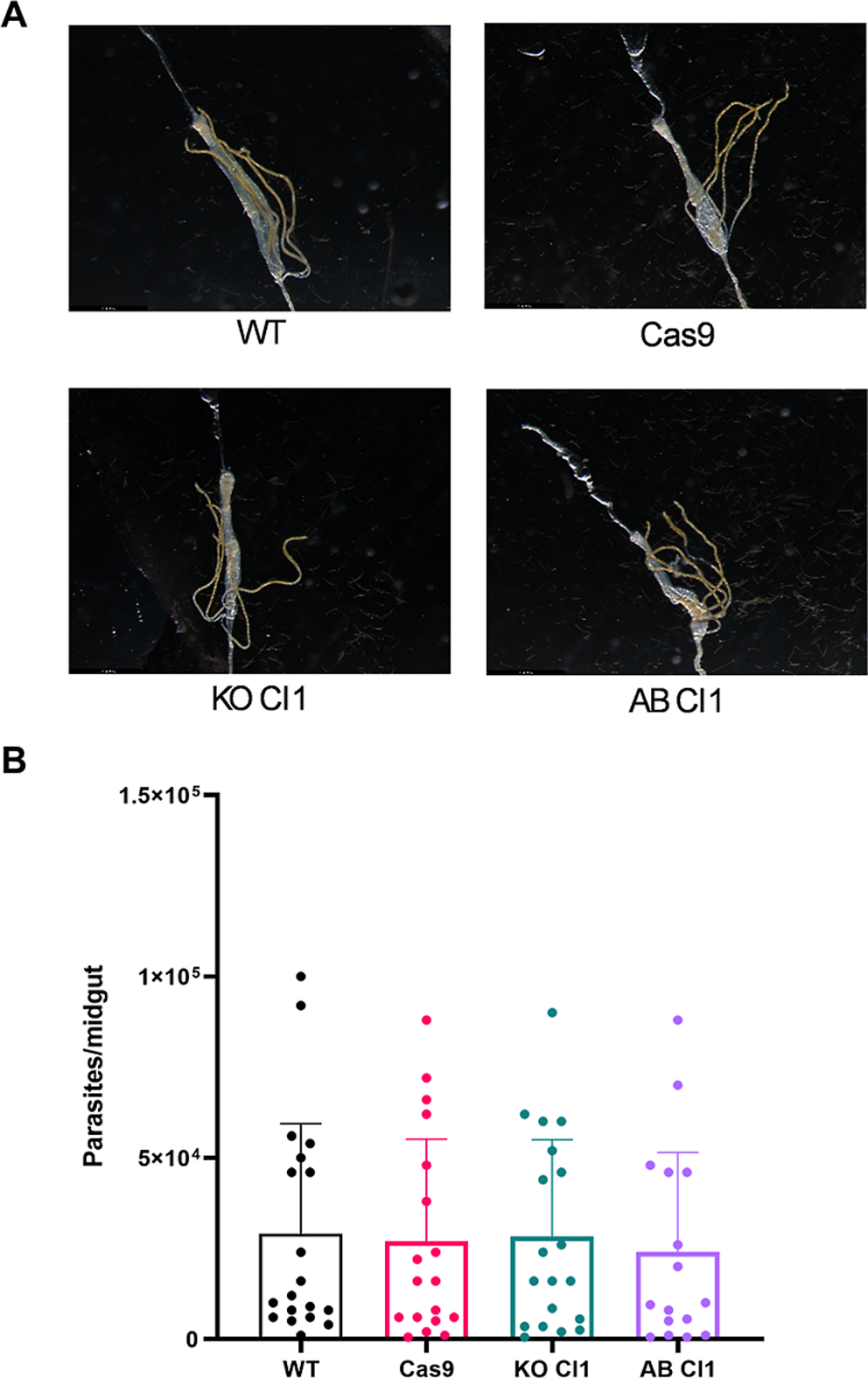
Establishment of WT, Cas9, KO, and AB lines in the sand fly midgut. *Lutzomyia longipalpis* sand flies were infected with each of the parasite lines via artificial membrane feeding. Midguts were dissected 7 days postblood meal and the infection burden was determined. (A) Representative images of midguts dissected from sand flies infected with WT, Cas9, KO Cl1, and AB Cl1 parasite lines are shown. Scale bars = 1.1 mm. (B) The total number of parasites per midgut of sand flies infected with WT, Cas9, KO Cl1, and AB Cl1 are also represented. Each symbol represents a single midgut. The group average and 95% CI are also shown. Statistical significance was determined using the Kruskal–Wallis test, followed by post hoc analysis with Dunn’s multiple comparisons test. No statistically significant differences were detected. Data are represented as the mean ± standard error of three independent experiments.

### Loss of LABCG1 Does Not Affect Parasite Virulence *In Vivo*

Finally, to assess whether the differences in infectivity could impact parasite virulence *in vivo*, BALB/c mice were infected with the WT PH8 strain as well as the mutant lines Cas9, KO Cl1, and AB Cl1. Lesion development was monitored for 9 weeks. Curiously, the lesions caused by the mutant strains (Cas9, KO Cl1, and AB Cl1) were smaller than those produced by the WT parasites (Figure 7A,B). Despite this, no difference in lesion size was observed among animals infected with each of the mutant strains, suggesting that the reduction may be a consequence of the Cas9 expression. In line with this, no differences in parasite load, as assessed by limiting dilution, were detected in the footpads of animals infected with the different mutant strains as well as with the WT control (Figure 7C). Altogether, these results suggest that the loss of LABCG1 does not affect parasite survival and multiplication *in vivo* at least in the context of our BALB/c infection model.

**Figure 7:**
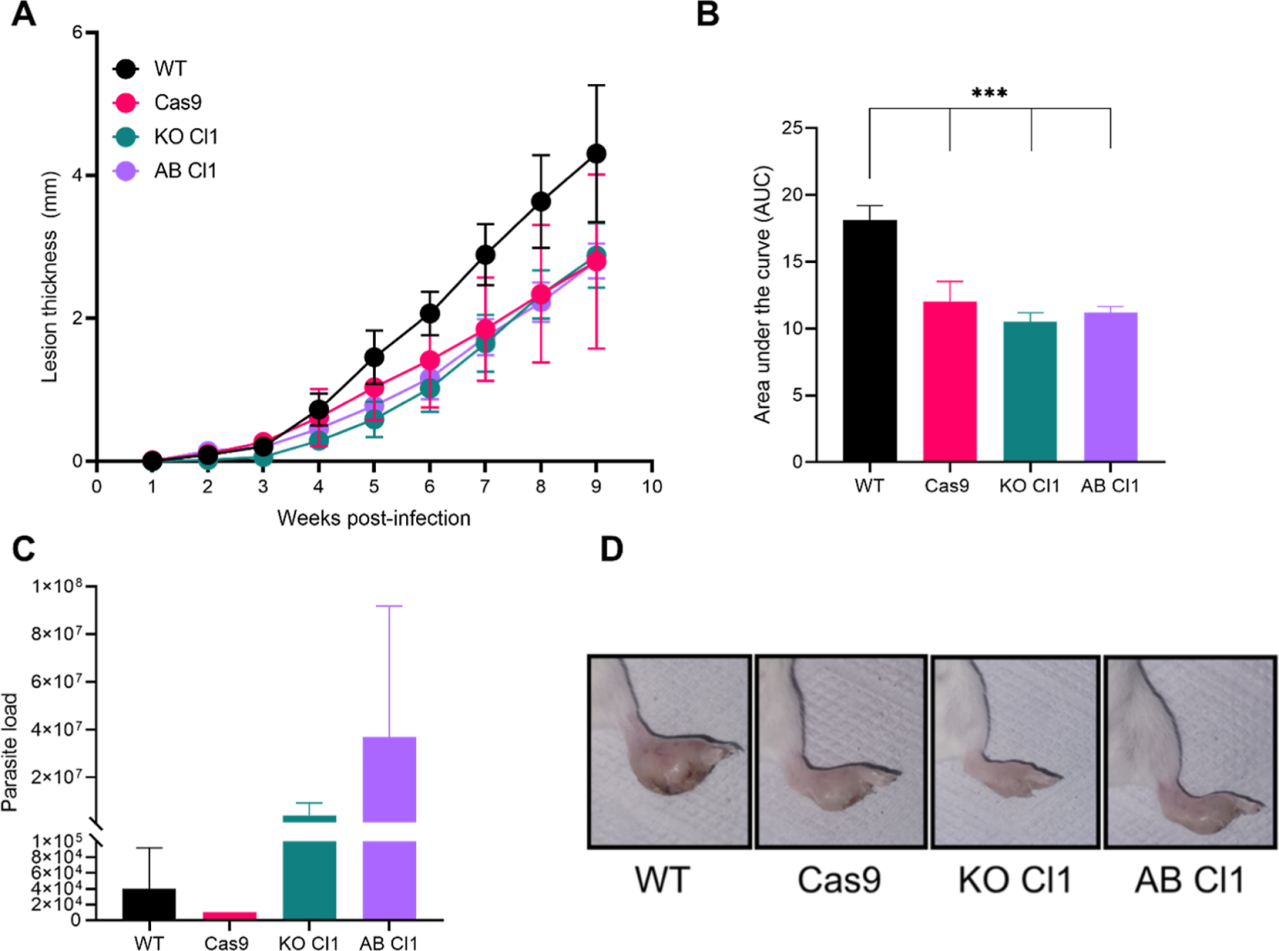
*In vivo* infection of BALB/c mice with WT, Cas9, KO, and AB lines. (A) Lesion thickness (mm) of mice (*n* = 5) infected with WT, Cas9, KO Cl1, and AB Cl1 parasites over 9 weeks. (B) The area under the curve shown in panel A. (C) Parasite load after 10 weeks of infection (*n* = 3). (D) Representative images of lesions 9 weeks postinfection. Graphs represent data from a single experiment. Statistical analysis using one-way ANOVA with Tukey’s post hoc test. Nonsignificant data are not shown. ***p ≤ 0.001. Panels A and B show representative results from two independent experiments with similar profiles, while the parasite load shown in panel C corresponds to a single experiment.

## Discussion

Evasion of the host immune system is a common strategy among obligatory parasites such as*Leishmania*, which over time have developed molecules capable of modulating various processes and ensuring the establishment of infection.^15^ These molecules are known as virulence factors and have been widely studied over the past decades.^15,30–34^ The importance of studies in this area is directly related to the development of new diagnostic methods, new therapies, and a better understanding of the particularities of *Leishmania* species.^15,35^ A previous work from our group suggested that ABCG1 could be a virulence factor due to its higher abundance in the more virulent *L. (L.) amazonensis* strain (PH8 versus LV79 strain).^16^ This hypothesis was supported by studies in which both LABCG1 and LABCG2 were knocked out in *L*. (*L*.) *major*, leading to a decrease in infectivity and virulence.^23^

To verify this hypothesis, we knocked out the *LABCG1* gene in PH8 and evaluated the impact of its absence on different parameters related to virulence. First, we showed that the loss of LABCG1 did not impact the proliferation of promastigotes in cultures. This was expected since no difference was observed between the proliferation of LV79 and PH8 strains.^16^ Furthermore, we did not observe differences in the metacyclogenesis profiles of the knockout line. This was not expected since PH8 and LV79 parasites have shown differences in metacyclogenesis, as did *L*. *(L.) major* when simultaneously knocked out LABCG1 and LABCG2.^16,23^ In the case of the *L. (L.) major* mutant, the decrease in metacyclics in culture was suggested to be linked to defects in autophagy and oxidative stress response, whereas the difference in metacyclogenesis of PH8 versus LV79 was likely due to a combination of factors unique to each strain.^16,23^

We also thought that the loss of LABCG1 could affect complement-mediated lysis since PH8 was previously shown to be more resistant to it than LV79 ^24^. Additionally, the double knockout of LABCG1 and LABCG2 in *L. (L.) major* impaired the parasites ability to survive complement-mediated activity.^23^ However, we observed that the loss of LABCG1 alone did not affect resistance to complement-mediated lysis that was comparable to that of its parental cell line. However, the add-back parasites, which express 41-fold more LABCG1 transcripts than wild-type parasites, were more susceptible to complement. We hypothesized that the overexpression of this transporter might have affected LPG exposure in the parasite membrane. Indeed, the overexpression of an ABCG in *L. (L.) infantum* impaired the uptake of phospholipid analogues.^36^ Since phospholipid content is closely associated with the presence of lipid rafts in the membrane, this could potentially influence LPG amount and directly its susceptibility to complement-mediated lysis.^37–40^ Unexpectedly, we observed that the LPG levels did not significantly differ between the mutants and the wild-type strain, suggesting that the increased complement susceptibility of the add-back parasites is likely due to a different mechanism that was not investigated in the present study. Of note, contrary to our results, in *L. (L.) major* lacking LABCG1 and LABCG2, others have observed considerable alterations in LPG composition.^23^ Taking all of the above in consideration, we thus believe that the LPG polymorphisms observed between LV79 and PH8 in our previous work are probably associated with a combination of factors that may not include the differential expression of LABCG1.^24^

Interestingly, our *in vitro* experiments demonstrated that the loss of LABCG1 impaired the infection ability of the parasites, which was recovered when the gene was added. Considering that there were no differences in phagocytosis, it is probable that parasites lacking LABCG1 have lower survival inside macrophages (as compared with control parasites). A reduction in infectivity was also observed in *L. (L.) major* parasites lacking both LABCG1 and LABCG2.^23^ However, while these double-knockout parasites also showed reduced virulence *in vivo*, in our study, the absence of LABCG1 did not impair parasite fitness or its capacity to generate lesions in a mouse model of infection. Likewise, we did not observe any differences in the ability to colonize sand flies between the wild-type PH8 and the mutant parasites, suggesting that LABCG1 is not essential for the establishment of *L. (L.) amazonensis* in the sand fly vector. Therefore, the difference in sand fly infection by the LV79 and PH8 strains we observed previously is likely independent of the differential LABCG1 expression detected in those strains.^24^ Of note, the discrepancy between our *in vitro* and *in vivo* experiments can be attributed to the complexity of the *in vivo* model, in which parasites interact with a variety of host/vector cells and immune components that we cannot easily (or even at all) reproduce *in vitro*.

A previous study in *L. (L.) major* shows that the simultaneous loss of LABCG1 and LABCG2 reduced parasite virulence, highlighting the importance of these transporters in host–parasite interactions. In contrast, our results demonstrate that LABCG1 alone is not essential for the virulence of the PH8 strain of *L. (L.) amazonensis*. Although its higher abundance in PH8 compared to LV79 initially suggested a role in parasite infectivity and virulence, our data demonstrates that other molecular factors are likely responsible for the enhanced virulence of PH8 strain, eventually including LABCG2, which may compensate for the absence of LABCG1. These findings highlight the specific roles of ABC transporters in *Leishmania* biology across different species.

## Conclusion

Loss of the LABCG1 transporter in *L. (L.) amazonensis* did not affect parasite growth, metacyclogenesis, LPG characteristics, or virulence in murine hosts and phlebotomine vectors. However, LABCG1 deficiency slightly reduced parasite infectivity *in vitro*, indicating a possible role for this transporter in macrophage–parasite interactions rather than in parasite *in vivo* establishment or transmission.

## Methods

### Ethical Statement

Four- to eight-week-old female BALB/c mice were purchased from the Animal facility of Faculty of Medicine, University of São Paulo. Mice were kept at the Animal facility of the Department of Parasitology and Microbiology, Institute of Biomedical Sciences, University of São Paulo, with sterile water and food ad libitum. All animal experiments were done in accordance with the norms from Conselho Nacional de experimentação Animal (CONCEA), and the protocols were approved by the Institutional Animal Care and Use Committee (CEUA) (Protocol N° 9829290419).

For the experiments conducted at the National Institutes of Health (NIH), all animal experiments were approved by the National Institute of Allergy and Infectious Diseases (NIAID) Animal Care and Use Committee under the LMVR4E animal protocol. The NIAID DIR Animal Care and Use Program complies with the Guide for the Care and Use of Laboratory Animals and with the NIH Office of Animal Care and Use and Animal Research Advisory Committee guidelines.

### Primer Design

Primers for CRISPR editing were designed using the LeishGEdit platform (http://www.leishgedit.net/) and aligned against the *L. (L.) amazonensis* genome assembly (strain UA301) deposited at the NCBI (accession ASM531712v1), using the SnapGene tool. The remaining primers were designed with SnapGene, based on the same genome assembly data (accession ASM531712v1). Table S1 lists the primers designed and used in this study.

### Quantitative Real-Time PCR

To analyze LABCG1 transcript levels, promastigotes (5 x10^7^) from each strain or lineage were collected by centrifugation and their RNA was extracted using TRIzol (Life Technologies) following the manufacturers’ instructions. cDNA was synthesized from 2.5 μg of DNase I (Thermo Fisher Scientific)-treated RNA using Oligo­(dT), random hexamers, and SuperScript II Reverse Transcriptase kit (Invitrogen) according to the manufacturer protocol. Real-Time PCR reactions were performed in a StepOne Plus thermocycler (Thermo Fisher Scientific) using the Maxima SYBR Green/Rox qPCR MasterMix (Thermo Fisher Scientific) and specific primers for coding sequences of LABCG1 and GAPDH (supp. Table 1). The PCR program consisted in 95^○^C for 10 min followed by 40 cycles at 95^○^C for 15 s, 60^○^C for 60 s, and 72^○^C for 20 s. The 2^–ΔΔCt^ method was employed to determine the relative expressions using GAPDH as the reference gene. Normalization was performed against LV79 day 2 for comparisons between PH8 and LV79, and against PH8 WT for comparisons among mutant strains. Three biological replicates per strain and three technical replicates per sample were analyzed.

Potential copy number variations in the LV79 and PH8 strains were determined by real-time PCR using genomic DNA (gDNA) from promastigotes. Standard curves were constructed using LV79 gDNA, ranging from 2 × 10^6^ to 2 parasites, considering that each diploid genome of *L. (L.) amazonensis* corresponds to approximately 0.07 pg of DNA. Ultrapure water was used as a negative control. Reactions were performed as described above, using 66 ng of gDNA and the same cycling conditions. The primers used in this assay are listed in Table S1 (LABCG1 RT F/R and G6PD RT F/R). The relative copy number of LABCG1 was calculated as the ratio between LABCG1 and G6PD quantities, both determined from the LV79 standard curve.

### Constructs for Knockout and Add-Back Generation

The knockout of LABCG1 was performed using the CRISPR-Cas9 technique. An *L. (L.) amazonensis* PH8 line expressing hSpCas9 and T7RNAP was obtained via transfection with the plasmid pTB007.

For amplification of the donor DNA, we used 0.5 μM of each primer (F KO LABCG1 and R KO LABCG1, Table S1), 200 μM of dNTPs (Invitrogen), 1 U of Platinum SuperFi II DNA polymerase (Invitrogen), 5× buffer, 100 ng of pTBlast or pTPuro plasmids,^26^ and DEPC water q.s.p. 100 μL. Amplification was performed in a Veriti thermocycler (Applied Biosystem) at 98 °C for 30 s, followed by 35 cycles of 98 °C for 10 s, 60 °C for 30 s and 72 °C for 2 min and 15 s, followed by a final extension step at 72 °C for 7 min. For amplification of the sgRNA, we used the 0.5 μM of each primer combination (G00 and sgRNA U KO LABCG1 or G00 and sgRNA D KO LABCG1, supp. Table 1), dNTPs and SuperFi II DNA polymerase, and DEPC water as described above. The amplification conditions used were 98 °C for 30 s, followed by 35 cycles of 98 °C for 10 s, 60 °C for 30 s and 72 °C for 30 s, followed by a final extension step at 72 °C for 5 min. The PCR products were precipitated by incubating with 0,1 volumes of 3 M sodium acetate and 2 volumes of ethanol at 20 °C for 10 min, followed by centrifugation at 16.000*g* for 10 min at 10 °C. The pellet was washed with ethanol 70% and centrifuged at 16.000*g* for 5 min at 10 °C and after drying was resuspended in 300 μL of transfection buffer (Tb-BSF (90 mM Na_2_HPO_4_, 5 mM KCl, 0.15 mM CaCl_2_, 50 mM HEPES, pH 7.3))

To obtain the add-back parasites, the LABCG1 gene was amplified by PCR using the primers add-back F LABCG1 and add-back R LABCG1 (supp. Table 1), which target the untranslated regions flanking the LABCG1 gene. Amplification followed the same PCR setup described above, and 5 μL of the reaction was analyzed by electrophoresis in a 1% agarose gel. Both the PCR product and the pSPαZEOα vector were digested with *Xba*I (Invitrogen) and *Hind*III (New England Biolabs) and ligated using T4 DNA ligase (Invitrogen) following the manufacturer’s instructions. The resulting construct pSPαZEOα/LABCG1 was transformed into *Escherichia coli* DH5α, ampicillin resistant clones were selected and growth in liquid culture, and plasmids extraction was performed using the Wizard Plus SV Minipreps DNA Purification System (Promega). The plasmid insert was confirmed by Sanger sequencing at the Genoma–IB facility of the University of São Paulo.

### Transfection by Electroporation

Promastigotes were transfected using the Amaxa Nucleofactor (Lonza). Briefly, 1 × 10^7^ logarithmic phase parasites were collected by centrifugation (1300xg for 10 min), washed twice with PBS, and resuspended in Tb-BSF in the presence of 5 μg of the plasmid (pT007 or pSP72αZEOα/LABCG1 construct) or donor DNA and sgRNA templates. For knockout, donor DNA and sgRNA were transfected into the PH8 line expressing hSpCas9 and T7RNAP. To generate the addback line, the pSP72αZEOα/LABCG1 construct was transfected into the LABCG1 knockout cell line (clone 1). Parasites were transferred to Gene Pulser cuvettes (Bio-Rad) and electroporated using the X-001 program configuration. The electroporated parasites were transferred to 5 mL of 199 medium (M199) supplemented with 10% of fetal bovine serum (FBS) and 20 μg/mL of gentamicin and incubated at 24 °C for 16 h, followed by the selection with the appropriate antibiotic (32 μg/mL hygromycin B for hSpCas9-T7RNAP lineage, 12 μg/mL blasticidin, and 20 μg/mL puromycin for the knockout line and blasticidin, puromycin, and 300 μg/mL zeocin for the addback line). The cultures were maintained at 24 °C until we observed parasite growth.

The knockout and addback transgenic lines were cloned by serial dilution in M199 supplemented with 10% FBS, 20 μg/mL gentamicin, and the antibiotics used for selection in 96-well plates. The plates were incubated at 24 °C for 15 days, after which three positive wells were subcultured in 25 cm^2^ flasks.

### Parasite Culture, Growth Curve, and Metacyclic-Enriched Purification

*Leishmania (L.) amazonensis* PH8 (IFLA/BR/1967/PH8) and LV79 (MPRO/BR/1972/M1841) wild-type strains were cultivated in vitro in M199 medium as described by Tano et al. (2022).^16^ Cultures were synchronized by three consecutive passages every 3 days starting from an inoculum at 5 × 10^5^ promastigotes/mL.^41^ Parasites were maintained under the appropriate antibiotics, and growth curves were determined by counting in a hemocytometer daily for 7 days. The Ficoll gradient method was performed to estimate the proportion of metacyclic promastigotes.^42^ Promastigotes were maintained by regular subculturing and used up to the eighth passage.

### Complement-Mediated Lysis Assay

Complement-mediated lysis was evaluated according to a previously published protocol with modifications.^43^ Briefly, 1 × 10^7^ early stationary phase promastigotes were incubated with RPMI medium containing 2% human serum for 30 min at 34 °C. The cell viability was assessed using 7-AAD labeling (Thermo Fisher Scientific) in a flow cytometer. Heat-killed promastigotes (64 °C for 30 min) and nonlabeled promastigotes were used as gating controls, and promastigotes not treated with human serum were used as the internal negative control.

A total of 100,000 events were recorded using the BD Accuri C6 Plus flow cytometer (BD Biosciences). Data were analyzed using FlowJo version 10.

### LPG Characterization

LPGs from procyclic promastigotes from PH8 strains (WT, Cas9, KO, and AB) and *L. (L.) infantum* (Ba262-control) were extracted in solvent E (H2O/ethanol/diethyl ether/pyridine/NH4OH; 15:15:5:1:0.017) and purified with phenyl-sepharose.^27^ Five μg of LPG was submitted to Western blot using the mAbs CA7AE and LT22 (1:1000). The former recognizes Gal­(β1,4)­Man­(α1)-PO_4_ repeat units^44^ and the latter recognizes β1,3-Glc side chains,^45^ respectively. To analyze repeat units, LPGs were depolymerized and subjected to mild acid hydrolysis (0.02 N HCl, 100 °C, 5 min).^46^ Polysaccharides were labeled with 0.05 N ANTS (8-aminonaphthalene-1,3,6-trisulfate) and 1 M cyanoborohydride (37 °C, 16 h), subjected to FACE, and the gel was visualized under UV light. G_1_-G_7_ oligo-glucose ladders were used as standards.^27^

### Macrophage Infection

Bone-marrow-derived macrophages (BMDM) were obtained from BALB/c mice as previously described.^47^ Four × 10^5^ BMDMs were plated on coverslips in 24-well plates, in RPMI medium with 10% heat-inactivated fetal calf serum and 20 μg/mL gentamicin and infected with early stationary-phase promastigotes at a ratio of 5:1 (parasites/macrophage).^24^ Nonphagocytosed parasites were removed by washing after 4 h, and macrophages were maintained in fresh RPMI medium for 24, 48, and 72 h at 34 °C with 5% CO_2_. Cells were fixed with methanol-PBS (1:1) and stained using the Instant Prov Kit (Newprov). The percentage of infected macrophages and the number of amastigotes per cell were determined by counting one hundred macrophages per coverslip. Three independent experiments with technical triplicates were performed.

### Adhesion and Phagocytosis of Promastigotes by Macrophages

BMDMs were incubated with early stationary phase promastigotes at a 5:1 ratio (parasites/macrophage), and the assay was performed as described elsewhere.^48^ Images were captured using an Axiovert. An A1 inverted fluorescence microscope (Carl Zeiss) was equipped with an Apotome 3 (Carl Zeiss) and analyzed using the ZEISS ZEN software version 3.7. A total of 100 macrophages were analyzed to quantify the number of attached (green and blue, labeled with Alexa 488 and DAPI) and phagocytized (blue, stained with DAPI) promastigotes.

### *In Vivo* Infection

Four- to eight-week-old female BALB/c mice (*n* = 5/group) were infected in the left hind paw with 2 × 10^6^ *L. (L.) amazonensis* promastigotes of the WT, Cas9/T7 (Cas9), ABCG1 knockout (KO), and add-back (AB) lines. Footpads were measured once a week with a digital caliper (Mitutoyo Corporation, Japan) to determine lesion sizes. After 9 weeks, animals were euthanized, and the parasite burden in lesions was determined by limiting dilution.^49^

### Sand Fly Infection

*Lutzomyia longipalpis* sand flies were mass-reared at the Laboratory of Malaria and Vector Research insectary in the NIH as previously described.^50^ Adult females were maintained on a 30% sucrose diet and were starved for 12 h before feeding. Sand flies were infected via artificial feeding through a chick membrane on heat-inactivated, defibrinated rabbit blood (Spring Valley Laboratories, MD, USA) containing promastigotes of *L. (L.) amazonensis* WT or transgenic lines used in this study (5 × 10^6^/mL), as previously described.^51^ After infection, blood-fed females were sorted and kept on a 30% sucrose diet. On the seventh day postinfection, sand flies were collected to assess the infection status.

Briefly, under a stereomicroscope, sand fly midguts were dissected in PBS and transferred to individual microtubes (Denville Scientific) with 50 μL of formalin solution (0.005% in PBS). Midguts were homogenized, and 10 μL were loaded onto disposable Neubauer chambers (Incyto). Slides were observed under a phase contrast microscope (Zeiss) at 400× magnification, and the total number of parasites per midgut was determined and recorded, as reported elsewhere.^52^ For representative purposes, fully intact midguts of sand flies infected with each *L. (L.) amazonensis* line were photographed under a Leica DFC 7000T stereomicroscope using the software Leica applications suite X v3.7.5.24914 and filmed under a phase contrast microscope (Zeiss) at 400× magnification, as previously described.^53,54^

### Data Analysis

The data were analyzed using Prism 8 software. Statistical significance was assessed using (i) for 2-group comparisons, the Shapiro–Wilk test, and *t*-test with Welch’s correction, (ii) for multiple comparisons, the One-way ANOVA test followed by Tukey’s post hoc analysis for parametric data, and (iii) the Kruskal–Wallis test followed by Dunn’s post hoc analysis for nonparametric data. Significance levels were set as follows: *p ≤ 0.05; **p ≤ 0.01; ***p ≤ 0.001; ****p ≤ 0.0001.

## Supplementary Material











## Notes

Primer sequences; amino acid sequence alignment of LABCG1 inset; in vitro infection of BMDMs by different parasite lines at multiple time points; and phagocytosis assay images of parasite lines in BMDMs (PDF)
Anterior midguts of infected *Lu. longipalpis* with each parasite line (MP4)
Anterior midguts of infected *Lu. longipalpis* (MP4)
Anterior midguts of infected *Lu. longipalpis* (MP4)
Anterior midguts of infected *Lu. longipalpis* (MP4)

## Abbreviations and acronyms

ABCG1: ATP-binding cassette subfamily G member 1
CRISPR-Cas9: Clustered regularly interspaced short palindromic repeats/CRISPR-associated protein 9
LPG: lipophosphoglycan
BMDM: bone marrow-derived macrophages
KO: knockout
AB: add-back
PCR: polymerase chain reaction
TL: tegumentary leishmaniasis
VL: visceral leishmaniasis
LCL: localized cutaneous leishmaniasis
MCL: mucocutaneous leishmaniasis
ADCL: Anergic diffuse cutaneous leishmaniasis
