# Supplementary material for: LABCG1 Affects Leishmania (Leishmania) Amazonensis Infectivity but Not Virulence or Sand Fly Infection
Source: ACS Infect Dis. 2026 May 7;12(6):1968–79. doi: 10.1021/acsinfecdis.6c00067 (PMC13270501; doi:10.1021/acsinfecdis.6c00067)
Supplement: Supplementary file 1 [file id6c00067_si_001.pdf]

LABCG1 affects *Leishmania (Leishmania) amazonensis* infectivity but not virulence or sandfly infection

Gustavo Rolim Barbosa[1], Gustavo Henrique Correa Soares[1], Taina Cavalcante[1], Pedro Cecilio[2], Rodrigo Pedro Soares[3], Paula Monalisa Nogueira[3], Fabiano Oliveira[2], Adriano Cappellazzo Coelho[4], Beatriz Simonsen Stolf[1]*

[1] Department of Parasitology, Institute of Biomedical Sciences, University of São Paulo, São Paulo, Brazil.

[2] Laboratory of Malaria and Vector Research, National Institute of Allergy and Infectious Diseases, National Institutes of Health, Rockville, MD, USA.

[3] René Rachou Institute, Oswaldo Cruz Foundation, Belo Horizonte, Minas Gerais, Brazil.

[4] Department of Animal Biology, Institute of Biology, University of Campinas, Campinas, SP, Brazil.

*E-mail: bstolf@usp.br

Supplementary Information

	**CRISPR primers**	
------------	Primer G00	**5'**aaaagcaccgactcggtgccactttttcaagttgataacggactagccttattttaacttgctatttctagctctaaaac **3'**
------------	Donor DNA F KO LABCG1	**5'**CGACCCTAGAGTCGTGCGGGCAGCGCGTCTgtataatgcagacctgctgc **3'**
------------	Donor DNA R KO LABCG1	**5'**AAGCGAAGCGGTGTCCTCACTTTCCCTCTGccaatttgagagacctgtgc **3'**
------------	sgRNA U KO LABCG1	**5'**gaaattaatacgactcactataggGGCAGCGAGTAGGATGTTTAgttttagagctagaaatagc **3'**
------------	sgRNA D KO LABCG1	**5'**gaaattaatacgactcactataggCCAGCCTTCACCACGGTACAgttttagagctagaaatagc **3'**
	**Primers for mutant confirmation**	
P3	Puro R	**5'**CGGCTTACGTGTCATGCACC **3'**
P4	Blast R	**5'**CCACACATAACCAGAGGGCAG **3'**
P1	LABCG1 UTR F	**5'**ACGCACAAGAGAGAAGAATGGA **3'**
	**Real-time RT PCR primers**	
------------	LABCG1 RT F	**5'**TTAGACAACGAACCGGCACAG **3'**
P2	LABCG1 RT R	**5'**GCGTTCTGGAGTCGAATTCAATGA **3'**
	G6PD RT F	**5'**CTTGTTGCCTCCGGCTAC**3'**
	G6PD RT R	**5'**GGCCATGTAAGCATCCTC**3'**
	**Add-back construction**	
P5	Add-Back F LABCG1	**5'**ATTTCTAGAACCTACTTGCCGCCGTCTT **3'**
P6	Add-Back R LABCG1	**5'**ATTAAGCTTTGCGATGTGAACCGACAGG **3'**

Table S1. Primer sequences.

Figure S1

```
Query: unnamed protein product Query ID: lcl|Query_125719 Length: 657

>unnamed protein product
Sequence ID: Query_125721 Length: 657
Range 1: 1 to 657

Score:1357 bits(3512), Expect:0.0,
Method:Compositional matrix adjust.,
Identities:656/657(99%), Positives:656/657(99%), Gaps:0/657(0%)

Query  1    MSRLDNEPAQNGITTDTEDQENISNDGHQPISSGERNPRLHGSIIEFDSRTPKADSVPNS
60
            MSRLDNEPAQNGITTDTEDQENISNDGHQPISSGERNPRLHGSIIEFDSRTPKADSVPNS
Sbjct  1    MSRLDNEPAQNGITTDTEDQENISNDGHQPISSGERNPRLHGSIIEFDSRTPKADSVPNS
60

Query  61   YSLPLSWHRLSYSVGKKRILCRLTGTALPGRCLAILGSSGAGKTTFLNAICDRLASGGKL
120
            YSLPLSWHRLSYSVGKKRILCRLTGTALPGRCLAILGSSGAGKTTFLNAICDRLASGGKL
Sbjct  61   YSLPLSWHRLSYSVGKKRILCRLTGTALPGRCLAILGSSGAGKTTFLNAICDRLASGGKL
120

Query  121  KLSGRRQLGDCEFERHFRKAMGFVAQDDIISPLSTPYDALWFSLRTRRGTSRAETAERVH
180
            KLSGRRQLGDCEFERHFRKAMGFVAQDDIISPLSTPYDALWFSLRTRRGTSRAETAERVH
Sbjct  121  KLSGRRQLGDCEFERHFRKAMGFVAQDDIISPLSTPYDALWFSLRTRRGTSRAETAERVH
180

Query  181  EVLNVLRLRHCCGTKVGIPGLEAGLSGGERKRCSIGIELICDPKILLLDEPTSGLDSVTS
240
            EVLNVLRLRHCCGTKVGIPGLEAGLSGGERKRCSIGIELICDPKILLLDEPTSGLDSVTS
Sbjct  181  EVLNVLRLRHCCGTKVGIPGLEAGLSGGERKRCSIGIELICDPKILLLDEPTSGLDSVTS
240

Query  241  AKVVHLLRQLSRTGRTMIYTIHQPTAEVLSYFDDVMLMTQGRIAYHGTMAASLDYFESIG
300
            AKVVHLLRQLSRTGRTMIYTIHQPTAEVLSYFDDVMLMTQGRIAYHGTMAASLDYFESIG
Sbjct  241  AKVVHLLRQLSRTGRTMIYTIHQPTAEVLSYFDDVMLMTQGRIAYHGTMAASLDYFESIG
300

Query  301  FPCPHKYTPTDYYMVLLQDSVTSNVLIKRWRKYLKNGPRTPHTAAVRLARSRSDSSAARF
360
            FPCPHKYTPTDYYMVLLQDSVTSNVLIKRWRKYLKNGPRTPHTAAVRLARSRSDSSAARF
Sbjct  301  FPCPHKYTPTDYYMVLLQDSVTSNVLIKRWRKYLKNGPRTPHTAAVRLARSRSDSSAARF
360

Query  361  LDAYIAKFGSSPAVQLYELTLRTMIEISRDSLYLFSYMAQAIFFAVVVGLIFLNVRDNVE
420
            LDAYIAKFGSSPAVQLYELTLRTMIEISRDSLYLFSYMAQAIFFAVVVGLIFLNVRDNVE
Sbjct  361  LDAYIAKFGSSPAVQLYELTLRTMIEISRDSLYLFSYMAQAIFFAVVVGLIFLNVRDNVE
420

Query  421  GVQDRQGVLFMTVMNRAMSSTFIMINTFNNARAVFMREQQAGAYSPLMFFLGRSFAEFPV
480
            GVQDRQGVLFMTVMNRAMSSTFIMINTFNN RAVFMREQQAGAYSPLMFFLGRSFAEFPV
Sbjct  421  GVQDRQGVLFMTVMNRAMSSTFIMINTFNNVRAVFMREQQAGAYSPLMFFLGRSFAEFPV
480

Query  481  QILAVLVESCILYWTVGLHHNPGSFFYYFGVIALLSQVATGLGFAISTSFPSLVVSSAVA
540
            QILAVLVESCILYWTVGLHHNPGSFFYYFGVIALLSQVATGLGFAISTSFPSLVVSSAVA
Sbjct  481  QILAVLVESCILYWTVGLHHNPGSFFYYFGVIALLSQVATGLGFAISTSFPSLVVSSAVA
540

Query  541  PLILIPLALGGGLFASTDRLRPYWYWLEKPSFMRHAYILVLRNELHNVHHIACDYYRWGD
600
            PLILIPLALGGGLFASTDRLRPYWYWLEKPSFMRHAYILVLRNELHNVHHIACDYYRWGD
Sbjct  541  PLILIPLALGGGLFASTDRLRPYWYWLEKPSFMRHAYILVLRNELHNVHHIACDYYRWGD
600

Query  601  GYCINQPRDGGTVMRLLGFDGDPQSASVYMWVSLVVMFFLLRSISVIALVVTAHEKM
657
            GYCINQPRDGGTVMRLLGFDGDPQSASVYMWVSLVVMFFLLRSISVIALVVTAHEKM
Sbjct  601  GYCINQPRDGGTVMRLLGFDGDPQSASVYMWVSLVVMFFLLRSISVIALVVTAHEKM
657
```

Figure S1. Amino acid sequence alignment of the LABCG1 insert. Alignment of the amino acid sequence of the LABCG1 insert cloned from *L. (L.) amazonensis* strain PH8, used in the construction of the pSPazeoa/LABCG1 vector, compared with the amino acid sequence of LABCG1 from the *L. (L.) amazonensis* isolate UA301, deposited at the NIH (ASM531712v1). The nucleotide sequences were translated using the Expasy Translate Tool (https://web.expasy.org/translate/). The red square indicates the mutated amino acid (substitution of valine by alanine). The alignment was performed using the NIH BLAST (Basic Local Alignment Search Tool) tool.

Figure S2

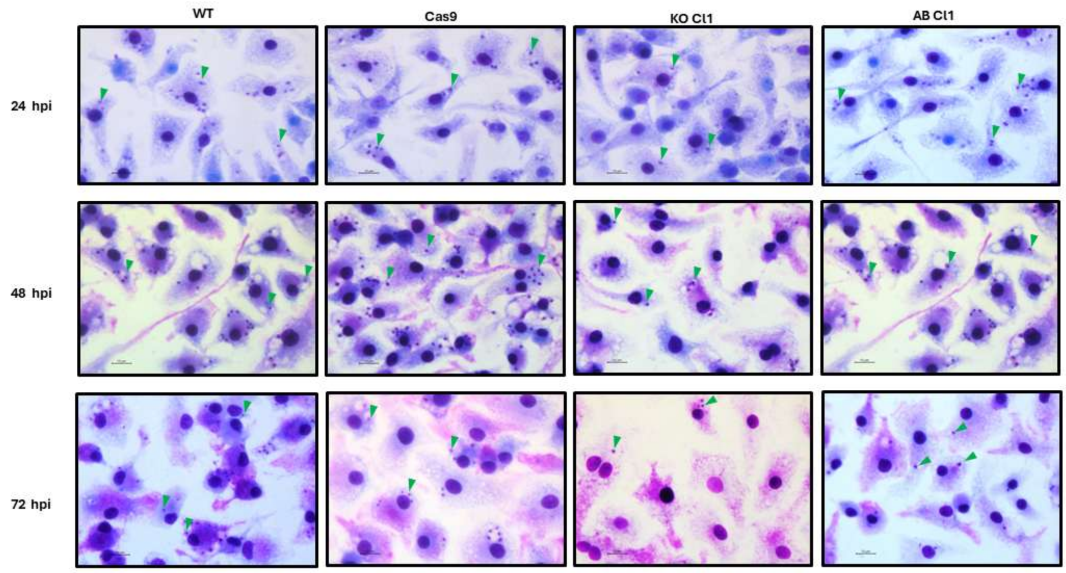

Figure S2. Representative images of the *in vitro* infection of bone marrow-derived macrophages (BMDMs) by *L. (L.) amazonensis* PH8 WT, Cas9, KO Cl1, and AB Cl1 at 24-, 48-, and 72-hours post-infection (hpi). (Figure 4); green arrows indicate amastigotes.

Figure S3

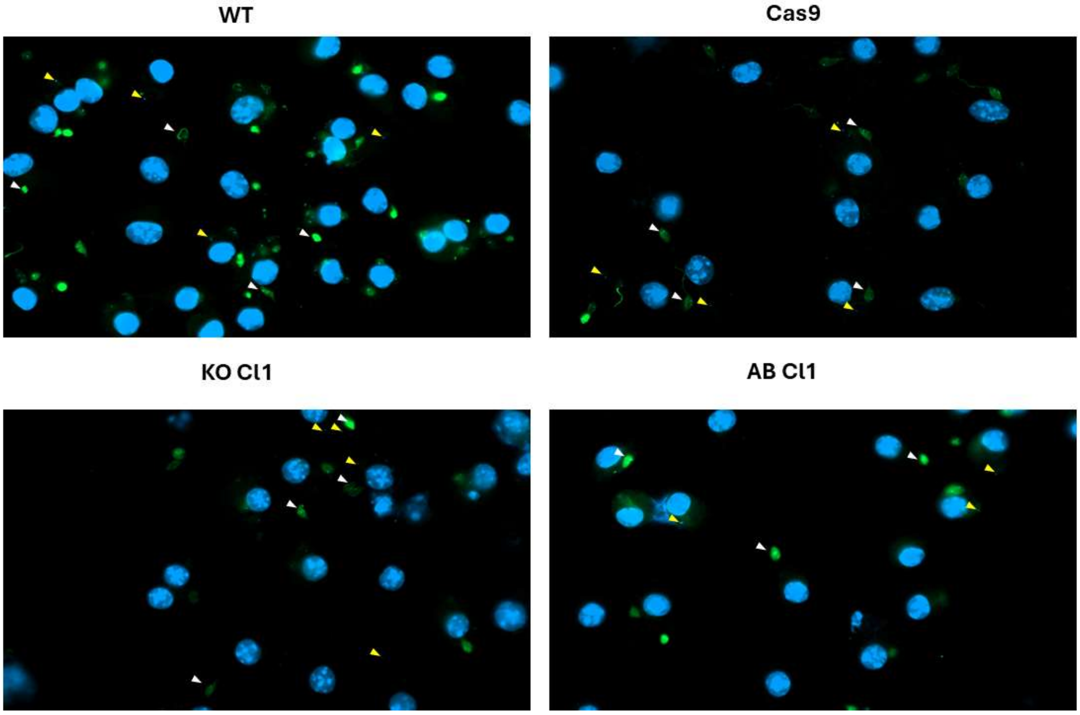

Figure S3. Representative images of the phagocytosis of WT, Cas9, KO Cl1, and AB Cl1 by BMDMs. White arrows indicate adhered promastigotes labeled with Alexa 488 (green), while yellow arrows indicate phagocytosed promastigotes labeled with DAPI (blue). The macrophage nuclei are stained with DAPI (blue). Images were acquired using a ZEISS Axio Observer 7 microscope with Apotome II and analyzed with ZEN lite software.

Video S1–S4. Anterior midguts of Lu. longipalpis infected with each parasite line (1- WT, 2- Cas9, 3- KO Cl1 and 4- AB Cl1).
